# Fatty acid specific δ^13^C values reveal earliest Mediterranean cheese production 7,200 years ago

**DOI:** 10.1371/journal.pone.0202807

**Published:** 2018-09-05

**Authors:** Sarah B. McClure, Clayton Magill, Emil Podrug, Andrew M. T. Moore, Thomas K. Harper, Brendan J. Culleton, Douglas J. Kennett, Katherine H. Freeman

**Affiliations:** 1 Department of Anthropology, The Pennsylvania State University, University Park, PA, United States of America; 2 Institute of Life and Earth Sciences, Heriot-Watt University, Edinburgh, United Kingdom; 3 Muzej grada Šibenika, Šibenik, Croatia; 4 Rochester Institute of Technology, Rochester, NY, United States of America; 5 AMS Radiocarbon Facility, Energy and Environmental Sustainability Labs, The Pennsylvania State University, University Park, PA, United States of America; 6 Department of Geosciences, The Pennsylvania State University, University Park, PA, United States of America; University of Illinois, UNITED STATES

## Abstract

The earliest evidence for cheese production in the Mediterranean is revealed by stable carbon isotope analyses of individual fatty acids in pottery residues from the Dalmatian coast of Croatia. Lipid residue data indicate the presence of milk in the earliest pottery, Impressed Ware, by 5700 cal. BCE (7700 BP). In contrast, by 5200 cal BCE (7200 BP), milk was common in refined Figulina pottery, meat was mostly associated with Danilo ware, cheese occurred in Rhyta, and sieves contained fermented dairy, representing strong links between specific function and stylistically distinctive pottery vessels. Genetic data indicate the prevalence of lactose intolerance among early farming populations. However, young children are lactase persistent until after weaning and could consume milk as a relatively pathogen-free and nutrient rich food source, enhancing their chances of survival into adulthood. Fermentation of milk into yogurt and cheese decreases lactose content. The evidence for fermented dairy products by 5200 cal BCE indicates a larger proportion of the population was able to consume dairy products and benefit from their significant nutritional advantages. We suggest that milk and cheese production among Europe’s early farmers reduced infant mortality and helped stimulate demographic shifts that propelled farming communities to expand to northern latitudes.

## Introduction

Current data on the transition to agriculture in Europe indicates an episodic spread of farming over ca. 3 millennia, starting about 7000 cal BCE. This expansion has been linked with the availability of milk and milk products as a calorie rich, potentially storable food source [1]. Milk production, previously considered a late phenomenon and part of a "secondary products" revolution [2], was in fact widely used by the earliest farmers in Europe and is documented through multiple lines of evidence including residues on pottery [3–5] and age-at-death data in ancient domestic animal populations [6,7]. In contrast, cheese production in the Mediterranean region is only documented beginning in the Bronze Age [8].

**Fig 1:**
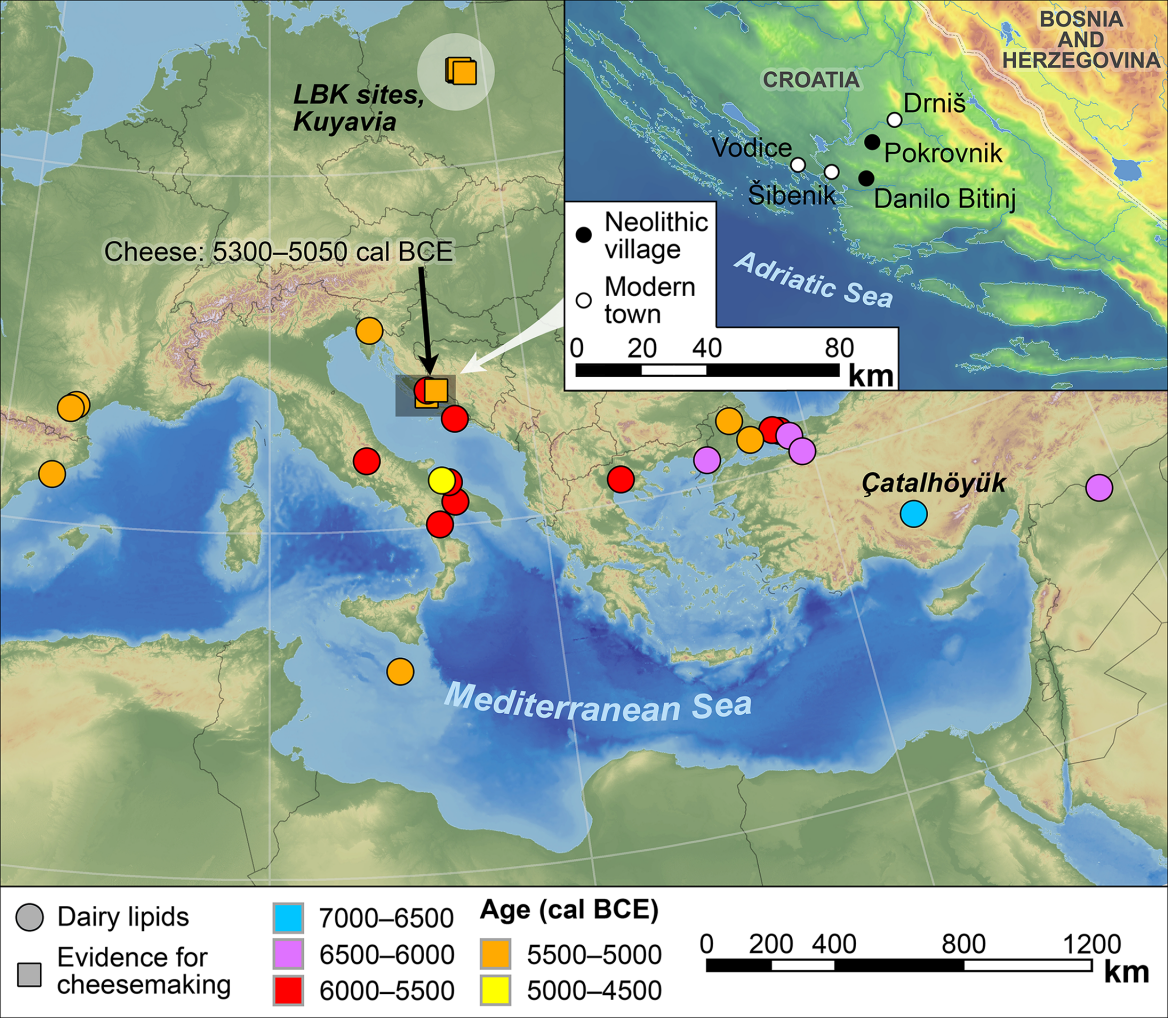
Location of Neolithic sites with direct residue evidence for dairying in the Mediterranean littoral and cheese in continental Europe [9,10]. Inset: the study area, showing the location of Pokrovnik and Danilo Bitinj on the Dalmatian coast of Croatia.

Here we report the earliest evidence for cheese production in the Adriatic through residue analysis of pottery sherds from two Neolithic village sites (Fig 1). Lipids document the presence of milk, meat and fish during the Early Neolithic (ca. 6000–5400 cal BCE) and the processing of milk into fermented products including cheese using distinctive pottery wares in the Middle Neolithic (beginning ca. 5200 cal BCE).

## Archaeological background

Archaeological data from northern Dalmatia indicate that farming with a full commitment to plant and animal husbandry as a subsistence system and sizable villages that persisted for centuries were already in place beginning 8000 years ago (e.g., [11–16]). Ongoing research on the earliest farmers of this region has resulted in a high-resolution chronology and detailed analyses of cultural and environmental proxies [3,12,14–26].

**Fig 2:**
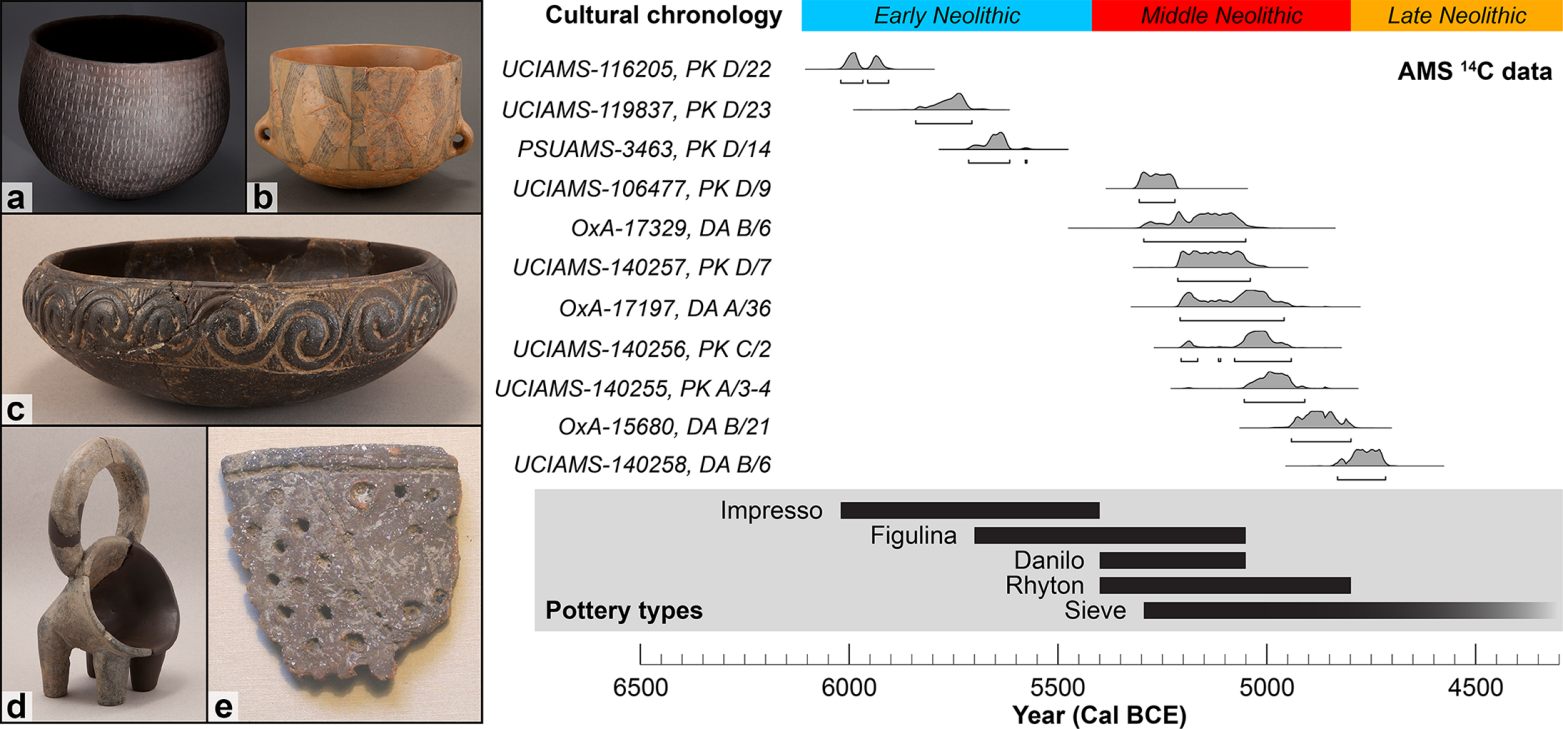
Probability distributions of calibrated ^14^C data of pottery types with associated residues from the sites of Pokrovnik and Danilo Bitinj and the general regional pottery chronology [14,17]. Pottery samples were selected from stratigraphic levels with radiocarbon dates generated on bones and seeds (see S1 Table for details). Examples of pottery types from the Dalmatian Neolithic: a. Impresso Ware; b. Figulina; c. Danilo fine ware; d. Rhyton; e. fragment of a sieve. Photos printed under a CC BY license, with permission from Muzej Grada Šibenika 2018.

Two villages, Pokrovnik and Danilo Bitinj, were occupied during the Early and Middle Neolithic (Fig 2; [15,16]). Recent excavations unearthed evidence of house structures, large quantities of animal bones, potsherds, stone tools, and other occupational debris. Radiocarbon dates indicate that Pokrovnik was continuously inhabited from 6000–5000 cal BCE, while current evidence for Danilo Bitinj places occupation at 5300–4800 cal BCE [13,14,25].

Pottery is a key artifact of early farming populations in the Adriatic. Early Neolithic farmers initially produced and used Impressed Ware pottery (starting 6000 cal BCE). This pottery is defined by impressed decorations on hand built, globular vessels with rounded bases and range from <5 cm diameter cups to large cooking or storage vessels exceeding diameters of 20 cm (Fig 2). Impresso pottery was made of local clays mixed with quartz or calcite temper, fired in open pits, and decorated with imprints from shells, fingernails, or animal teeth. Chronologically and stylistically similar wares are associated with the earliest farmers throughout the central and western Mediterranean.

Within a few centuries, Middle Neolithic potters began making a new suite of pottery styles with different decorative and technological methods. The presence of increasing technological refinement in Danilo ceramics defines Middle Neolithic (5400–4900 BCE) pottery in Dalmatia (Fig 2). Typical Danilo pottery included a wide variety of shapes including more open forms such as plates and bowls in addition to the globular jars. Fine wares were smudged, burnished, and decorated with incisions, often in banded ornamentation motifs of interlocking spirals and chevrons. Danilo wares were also made with local clays and fired in open pits, often in a reducing atmosphere. In contrast to Impressed Wares, typical Danilo pottery is distributed more locally in Dalmatia and the northeastern Adriatic.

Three subtypes of pottery are found in the Middle Neolithic. First, figulina, a less common and more refined pottery type, represents up to 5% of pottery assemblages. This ware was crafted by careful processing of local clays to create fine-grained textures. Vessels were fired at high temperatures in oxidizing atmospheres to produce buff-colored wares that were often slipped and painted in decoration [26]. Second, the highly distinctive rhyta, which are common in Middle Neolithic sites but represent a much smaller proportion of the pottery assemblages, are found throughout the Balkans [19,27]. These footed vessels have a large globular opening to the side and distinctive handles (Fig 2). Often zoomorphic or anthropomorphic in shape, these vessels were usually decorated on all surfaces with fine geometric incisions and fired in reduced or oxidized atmospheres. They were often partially painted and many of the incised decorations have white or red incrustations that highlight the motifs. Finally, the third type of pottery, sieves, are found in Dalmatia from the Middle Neolithic onwards, and are widespread in early farming communities throughout Europe. These vessels tend to be undecorated coarse wares with large numbers of holes punched into the walls (Fig 2).

The shifts in pottery form and refinement though time may have been accompanied by either significant changes or specialization in how the types of ware were used. The newly collected and predominantly unwashed potsherds from two Neolithic sites with long occupations prove a unique means to evaluate the co-development of form and function. Here, residue analyses from the different pottery styles sampled across the occupation series are used to elucidate the emerging importance of dairying by early farmers in Dalmatia. This progression suggests shifting dietary factors may have fostered the spread of farming from the Balkans into central Europe.

## Sample description

Potsherds from Pokrovnik (n = 27) and Danilo Bitinj (n = 20) consisted of Impressed ware (n = 10), figulina (n = 9), typical Danilo ware (n = 20), rhyta (n = 4), and sieves (n = 4). All samples were collected during recent excavations [14–16]. The Impressed ware, Danilo, and figulina samples were selected from the unwashed portion of the pottery assemblage from each site. Since rhyta and sieves are relatively rare in pottery assemblages, we selected samples of these pottery types from previously washed assemblages.

AMS radiocarbon dates were determined using bone and carbonized seeds from Pokrovnik and Danilo Bitinj in order to constrain the ages of associated pottery samples. Domestic animal bone samples (n = 8) were prepared in The Pennsylvania State University’s Isotope and Human Palaeoecology laboratory following methods outlined in McClure et al. [28,29] and Kennett et al. [29] and measurements were made at the UC Irvine Keck AMS Radiocarbon Facility. The University of Oxford Radiocarbon Accelerator Unit dated two carbonized seeds (Fig 2; S1 Table). Dates were calibrated with OxCal v 4.2.3 [30] using the IntCal13 atmospheric curve [31].

## Results

**Fig 3:**
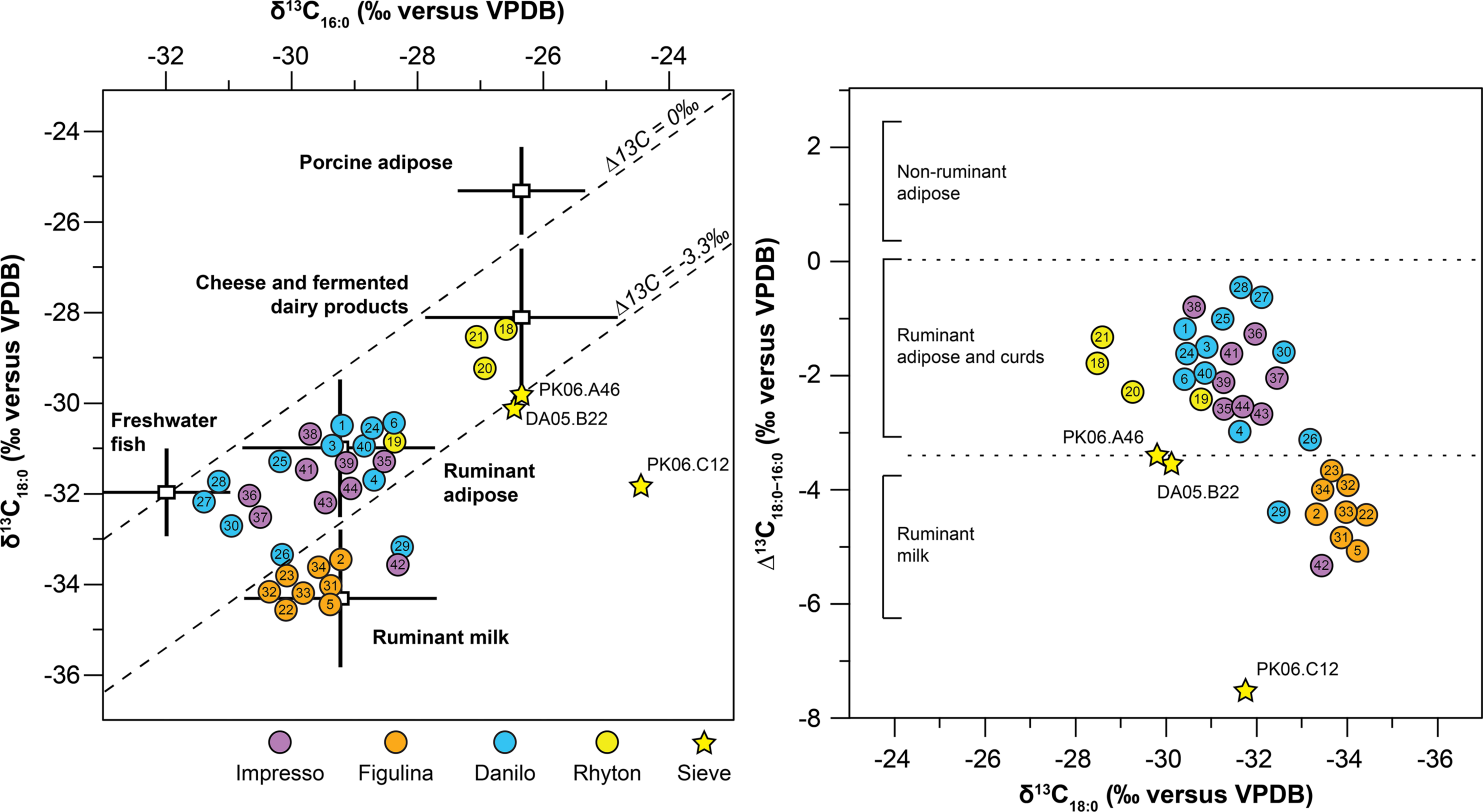
Stable carbon isotope compositions of individual fatty acids in lipid residues of animal origin from Neolithic potsherds from Pokrovnik and Danilo Bitinj, Dalmatian coast, Croatia. Animal origins are indicated based on previous studies by Evershed and colleagues [32,33,36,51]. (See S2 Table for list of pottery samples and biomarker data).

**Fig 4:**
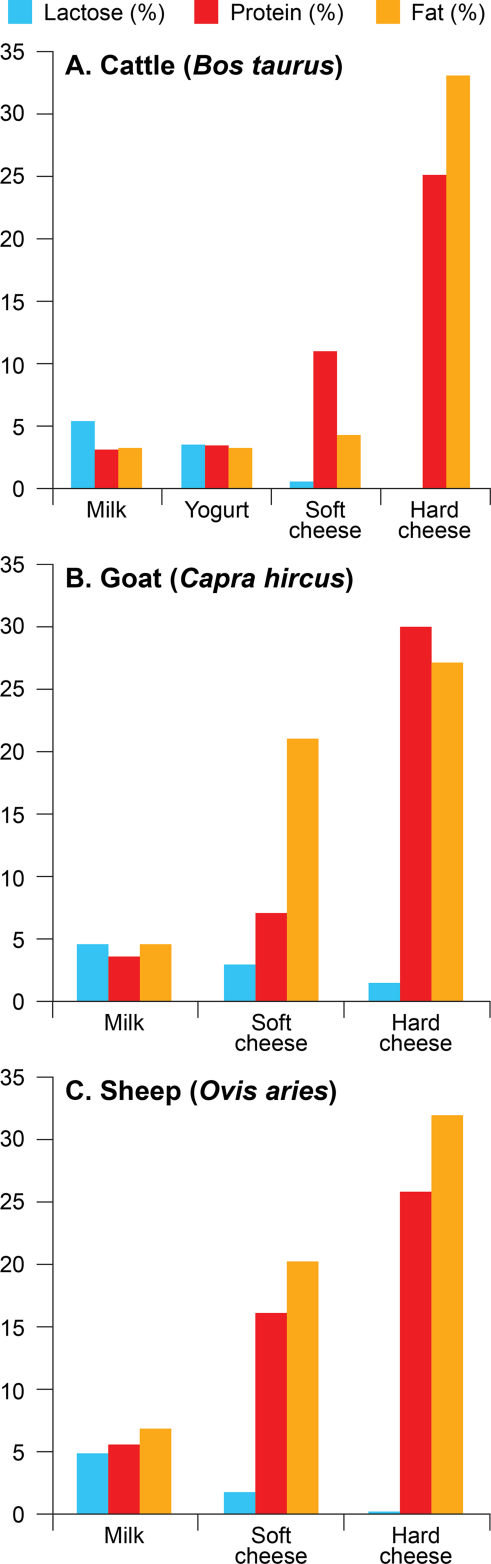
Differences in % lactose, protein, and fat. A. cattle (*Bos taurus*); B. goat (*Capra hircus*); and C. sheep (*Ovis aries*) dairy products [59,64]; see S3 Table for details.

Of the 47 potsherds, 36 (77%) yielded identifiable biomarkers in concentrations greater than 5 μg of lipids per gram of sherd (S2 Table; Figs 3 and 4). Overall lipid abundances were higher than those reported for other studies in the Mediterranean, and we suggest this was due to the use of recently excavated and unwashed samples [9]. Following the approach of Evershed et al. [5,32,33], palmitic and stearic fatty acids (16:0 and 18:0, respectively) were used to distinguish lipids derived from ruminant meat and milk fats, as well as cheese and fermented dairy products. Dietary sources of these lipids are distinguished by characteristic *δ*^13^C value quadrangles defined by *δ*^13^C_18:0_ versus *δ*^13^C_16:0_ values, as well as the isotopic difference between the two, [Δ^13^C_18:0–16:0_; [34]. Reference quadrangles were adopted from earlier studies of modern animal fats in southeast Europe [10,35–40] that had C_3_-dominated diets, since previous vegetation reconstructions suggest that Dalmatia was dominated by C_3_ plants during the early-to-mid Holocene [41]. Specifically, ruminant milk lipids are about 2 permil depleted in ^13^C relative to ruminant adipose lipids, while lipids in cheese and other fermented dairy products are 1–2 permil enriched in ^13^C relative to adipose lipids, at least when derived from ovicaprines [36,40,42]. Although the specific mechanism for apparent fractionation in cheese and fermented dairy products as compared to raw milk remains uncertain [36], there is some evidence to suggest that it is a consequence of the whey proteins denaturation [43] that lead to disulfide bonds among proteins, which then stabilize associated fat globules [44]. An alternative mechanism might be disproportionate conversion of (poly)unsaturated fatty acids (e.g., linoleic acid [C18:3]) to saturated homologues amid bacterial hydrogenation in rumens [45,46]. In either case, an apparent 1–2‰ would be expected [44,47–50].

Residue lipid isotopic data show clear chronological and technological patterns (Fig 3). Crossplots of *δ*^13^C_18:0_ versus *δ*^13^C_16:0_ values (left panel) delineate lipid residues derived from ruminant and non-ruminant dietary resources [5,32,33,36,51]. Crossplots of *δ*^13^C_18:0_ versus Δ^13^C_18:0–16:0_ (right panel) further show differences among lipid residues from ruminant adipose and secondary dairy products (i.e., cheese; 0‰ > Δ^13^C_18:0–16:0_ > -3.3‰) and from unfermented ruminant dairy fats (i.e., milk; Δ^13^C_18:0–16:_0 < -3.3‰) [36,52,53].

Most of the Early Neolithic Impressed Wares contained lipids that indicate ruminant adipose fats, although a smaller number contained signatures that suggest inputs from freshwater fish, despite the lack of fish bone in the faunal assemblages. Notably, one Impressed Ware vessel (#42) had signatures consistent with unfermented dairy fats, and dates to 5715–5576 cal BCE.

Residue lipids in samples of Middle Neolithic wares (5400–4900 cal BCE) indicated a significant diversity of likely origins. Further, we found clear functional distinctions between ceramic type: all of the buff, high-fired figulina wares (n = 8) contained unfermented ruminant dairy fats, while all but two of the coarser Danilo wares had primarily ruminant adipose and freshwater fish signatures. Three of the four rhyta tested had residues for secondary dairy products, consistent with cheese (0‰ > Δ^13^C_18:0–16:0_ > -3.3‰). This is the earliest documented lipid residue evidence for fermented dairy in the Mediterranean region and among the earliest documented anywhere to date [10].

In cheese production, the water-soluble lactose in liquid whey is strained from enzymatically treated or acidified coagulated milk curds, often using a coarse textile or a ceramic strainer [10]. Lipid residues from three of the four sieve samples were consistent with their use in the processing of ruminant milk into cheese or other fermented dairy products.

## Discussion and conclusions

This study presents the earliest evidence for cheese production in the Mediterranean region, providing clear data on the antiquity of milk in the early Neolithic and shifting technological practices in the Middle Neolithic linked to the production of cheese. This temporal pattern of emergent cheese production in a single region and at a single site, Pokrovnik, is currently unique. Previously, evidence of cheese production in the Mediterranean region was based on interpretations of land use patterns such as upland pastoralism, artifacts such as Italian “milk boilers” in the Bronze Age (ca. 1200 BCE) or bronze “cheese graters” from the 9^th^ century BCE, and literary evidence such as references to cheese in Homer’s Book XI of the *Iliad* [8].

Ancient DNA analysis indicates that Early Neolithic European farming populations did not possess the lactose tolerance allele that is found in subsequent populations beginning ca. 5000 years ago [54–56]. The enzyme lactase phlorizin hydrolase (LPH; or lactase) allows young mammals to digest milk by breaking down lactose, the main carbohydrate in milk. As mammals are weaned, lactase production generally declines [57,58]. In human young, lactase persistence ranges widely from weaning until much later in childhood development [up to >10 years of age; [59].

Consumption of milk and dairy products would have had many advantages for early farming populations. Milk, yogurt, and cheese are a good source of calories, protein, and fat. They could provide a reliable food between harvests or during droughts, epidemics, or famines [57]. Milk is a relatively pathogen-free source of fluids that could be critical during times of water scarcity [60]. Cheese provides a means of storing these nutrients to be used when milk production is low, and can be easily transported. Furthermore, fermentation of milk into yogurt or cheese lowers lactose content and allows lactose intolerant individuals to reap the benefits, while maintaining, or in some cases enhancing, other essential nutrients such as fat and calcium (Fig 4; S3 Table).

The benefits of milk, yogurt, and cheese consumption may have been especially important for children [56,61]. Early childhood is one of the most dangerous periods in pre-industrial human societies, as evidenced by increased mortality seen in numerous prehistoric skeletal collections [57,62,63]. The availability of milk or dairy products as a high calorie, pathogen-safe, and nutrient rich source of food for young children recently weaned likely helped those high-risk populations survive childhood.

Lipid residue data indicate farmers at Pokrovnik used milk for ca. 500 years before evidence of fermentation and cheese production, which emerged with an associated ceramic technology of functional differences in pottery manufacture and use. Dairying is well documented elsewhere in the Balkans [5] and indicates a persistent use of milk by the first farmers of Europe. The availability of milk in the Early Neolithic provided a source of digestible calories and low-pathogen liquids suitable for young children, potentially increasing their survivorship rates during a high mortality risk period. Milk also allowed young children to be weaned earlier, which in turn would have decreased birth intervals. This combination of increased childhood survival with increased birth rates would help explain the significant demographic transition noted for the Neolithic in Europe [65]. Furthermore, researchers link the antiquity of dairying to changes in the human genome and the emergence of lactase persistence as a case of niche construction [57]. Current distributions of lactase persistence are the result of selection over long periods of time in dairying communities. Children in early farming populations who were able to digest milk and tolerate dairy products into early adulthood would have had survival and reproductive advantages over other individuals.

The evidence for cheese making in the Dalmatian Middle Neolithic is contemporary with the first farmers of central Europe (Linearbandkeramik or LBK) and cheese production is documented in both regions [10]. This chronology indicates that fermented milk products were established dietary supplements by 5300 cal BCE and part of the agricultural package as farming spread into central and northern Europe. Current evidence in the Balkans suggests the scale of dairying and the target species changed during the Neolithic from a focus largely on goats to more cattle and sheep milk later in time as seen in both ceramic lipid analyses and zooarchaeological datasets that reconstruct mortality profiles for domestic animals [6].

Milk and associated products are also documented among the earliest farmers in other parts of central and northern Europe, based largely on cattle [1]. This spread of farming throughout Europe is associated with demographic shifts, beginning with the appearance of the LBK around 5400 cal BCE. Rowley-Conwy [1] hypothesized that the availability of fermented dairy products provided early farmers with a risk-buffering mechanism as a calorie rich, potentially storable food source. We suggest that dairying and fermentation had additional human life-history dependent advantages by reducing infant mortality. This helped stimulate demographic shifts that propelled farming communities to expand and provided the demographic and dietary risk buffering to allow Neolithic farming to spread to colder, temperate climates.

## Materials and methods

Artifacts and animal bones from Danilo Bitinj are deposited at the Muzej Grada Šibenika in Šibenik, Croatia, and from Pokrovnik at the Muzej Grada Drniš, Drniš, Croatia, and permission for analysis was granted by these institutions. Animal bone samples for AMS radiocarbon dating were selected from levels with pottery samples, and consist of domestic species. Animal bones were identified to element and species at the Šibenik City Museum and the Drniš City Museum, Croatia using a modern comparative collection and following standard identification procedures as outlined in Reitz and Wing [66]. Sample numbers and provenance information are listed in S1 Table. These samples were prepared following standard laboratory techniques at the Penn State University Human Palaeoecology and Isotope Geochemistry Laboratory and analyzed the University of California Irvine Keck Carbon Cycle AMS Facility [28,29]. Bone collagen was extracted and purified using a modified Longin [67] method with ultrafiltration [68]. As detailed in Kennett et al. [29], collagen samples were combusted for 3 h at 900 C in vacuum sealed quartz tubes with CuO and Ag wires and then reduced to graphite. Graphite samples were pressed into targets in Al cathodes and loaded on the target wheel for AMS analysis. Sample quality was evaluated with %C, %N and C:N ratios before further analysis [69,70]. Results were corrected for isotopic fractionation [71] and calibrated with OxCal 4.2 [72].

**Fig 5:**
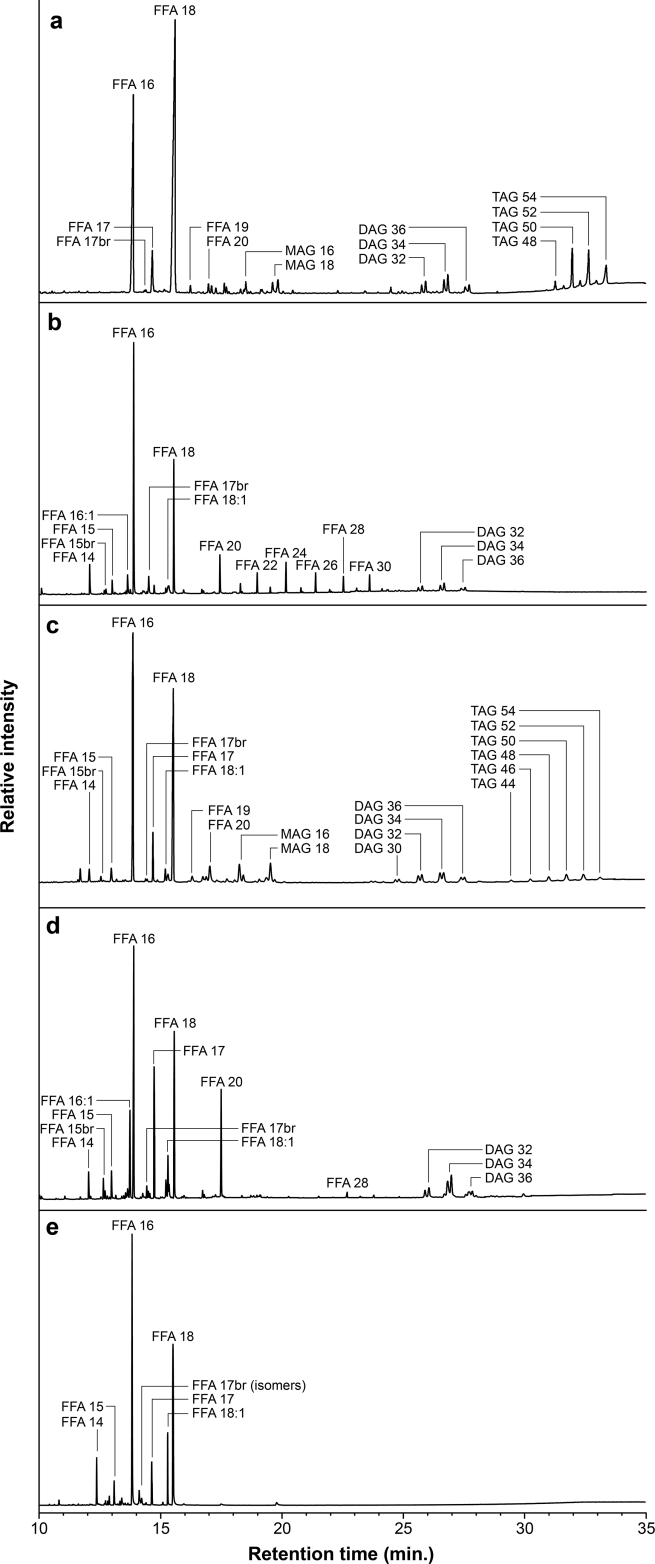
Partial total ion chromatograms (TICs) of lipid extracts derived from representative ceramic vessels at Neolithic sites on Croatia’s Dalmatian coast. A, Typical Danilo ware vessel that has molecular distributions indicative of degraded animal (ruminant) fats, such as a narrow distribution of triacylglyerols. B, Impresso style vessel that has molecular residue indicative of degraded fats vis-a-vis animal tissue and leaves. C, Figulina style vessel with molecular distributions indicative of dairy products, such as a wide distribution of triacylglyerols. D, Sieve with typical fermented dairy product lipid distributions, such as abundant branched fatty acids. E, Rhyton vessel that has molecular distributions indicative of firm cheeses, such as shorter-chain fatty acids. br, branched; DAG, diacylglyerol; FFA x:y, free fatty acid that has x carbon atoms and y unsaturations (i.e., double bonds); MAG, monoacylglycerol; TAG, triacylglycerol.

Residue analyses were conducted on potsherds excavated from two Neolithic sites in Dalmatia (Fig 5; S2 Table) following previously published techniques [5] (S4 Table). To summarize, every potsherd was abraded with a Dremel tool to remove surficial lipids and then rinsed with dichloromethane (DCM). Abraded and rinsed potsherds were extracted in an Accelerated Solvent Extraction system (ASE 200) with DCM:methanol (9:1 v/v) for 3 cycles of 5 minutes at 100°C. Resultant total lipid extracts were evaporated to dryness under nitrogen and then reconstituted in 200 uL of the extraction solvent for chromatographic separation in modified ASE 200 extraction cells [c.f., [73]. In short, the reconstituted total lipid extracts were separated into compound fractions defined by polarity and (un)saturation via selective sequential elution over silica gel with hexane (saturated hydrocarbons), hexane:DCM (85:15 v/v; unsaturated hydrocarbons), and DCM:methanol (70:30 v/v/; polar and functionalized lipids [e.g., general fatty acids]). Previous studies indicate such an approach delivers an efficient balance of high average recoveries (~90%) together with clean separation of functionalized lipids [73] for compounds such as alkanoic acids with high retention factor (*k*) values over silica with hexane and DCM (0.15 < *k* <0.75) [74].

Since saturated and unsaturated fatty acids are eluted together over silica with DCM:methanol, functionalized lipids in associated fractions were derivatized with N,O-bis(trimethylsilyl) trifluoroacetamide (BSTFA) of known isotopic composition (-47.9 ‰) before another chromatographic separation over silver-impregnated (5% w/w) silica gel with hexane (saturated fatty acids [e.g., C_16:0_ and C_18:0_]) and DCM (unsaturated fatty acids). Because milk fat is rich in acylglycerides, aliquouts of the methanol fractions (underivatized) furthermore were reacted with methanolic sodium hydroxide solution (5% *v*/*v*) for about 60 min at 70°C. Upon acidification with hydrochloric acid, hydrolyzed lipids were extracted as ‘free’ fatty acids into DCM before evaporated to dryness under nitrogen and derivatization with BSTFA. Note silylation was used to derivatize oxygen-containing groups (as opposed to methylation) because associated lipids also will be used for molecular radiocarbon analysis, which means a removable derivatizing group is preferable to a more stable derivatizing group (i.e., methyl) because (1) we still lack comprehensive radiocarbon analyses of the derivatizing groups in either case and (2) this approach circumvents any potential issues with transesterification [75].

Derivatized lipids of the saturated fraction were characterized first by gas chromatography mass spectrometry (GC-MS) with a Hewlett-Packard 6890 series GC and Hewlett-Packard 5973 mass selective detector. Samples were injected in splitless mode onto a 60-m DB5 fused-silica column (0.32 mm x 0.25 μm) via a Hewlett-Packard 7683 series autosampler. GC temperature was programmed to 60°C for 1 min then ramped to 350°C at 6°C·min^−1^ and held at final temperature for 20 min. Injector and detector temperatures were held at 350°C. Functionalized lipids (e.g., C_16:0_ and C_18:0_) were detected as trimethylsilyl (TMS) derivatives.

Isotopic signatures were characterized by gas chromatography-combustion-isotope-ratio monitoring mass spectrometry with a Varian 3400 modelGC connected to a Thermo MAT 252. Samples were injected in splitless mode onto a 60-m DB5 fused-silica column (0.32 mm x 0.25 μm) before combustion over nickel and platinum wire with oxygen in helium at 1,000°C. Values were determined relative to reference gas calibrated to VPDB and expressed in permil (‰) units: *δ*^13^*C* = 1,000(*Rsample/Rstandard* − 1),*R* = 13*C*/12*C*.

Within-run precision (1σ) and accuracy were determined by coinjected internal standards and are equal, respectively, to 0.10‰ and 0.11‰ (*n*-alkanes, *n* = 65). Standards were calibrated against the international reference materials NBS-22 [mineral oil] and IAEA-CH-7 [polyethylene foil]). Isotopic corrections for carbons added by BSTFA were made via measurements of the δ^13^C of benzene-1,2-dicarboxylic acid [commonly called phthalic acid (Schimmelmann standards)] after derivatization and then correcting for the mass-balanced δ^13^C value of derivatization carbon molecules.

## Supporting information

S1 Table AMS radiocarbon samples. (PDF)

S2 Table List of pottery samples and biomarker data. EN = Early Neolithic; MN = Middle Neolithic. Note: Pokrovnik Trench D level 2 and 3 are from one chronological stratum; Pokrovnik Trench D levels 5, 6, 7, and 8 are all from one chronological stratum [16]. Radiocarbon dates were calibrated with OxCal 4.2 [72].
(PDF)

S3 Table Average composition of cattle, sheep, and goat milk and products based on data summarized in [76–79]. (PDF)

S4 Table Summary of molecular and isotopic criteria to distinguish among animal and plant fats associated with archaeological artifacts and soils [10,35,38,53,55,80–88]. (PDF)
