# Supplementary material for: Fatty acid specific δ13C values reveal earliest Mediterranean cheese production 7,200 years ago
Source: PLoS One. 2018 Sep 5;13(9):e0202807. doi: 10.1371/journal.pone.0202807 (PMC6124750; doi:10.1371/journal.pone.0202807)
Supplement: S1 Table — (PDF) [file pone.0202807.s001.pdf]

Supporting Information

S1 Table. AMS radiocarbon samples

Site; Sample #	Trench/ Level	Lab #	Type	Species	Date BP	Date cal. BCE	Reference
Danilo	A/36	OxA-17197	Charred grain	*Triticum monococcum*	6121 +/-37	5210-4955	14
Danilo DA-16	B/6	UCIAMS -140258; PSU-6008	Bone	*Capra hircus*	5900 +/-25	4831-4716	
Danilo	B/6	OxA-17329	Charred seed	*Rosa sp.*	6204 +/-38	5295-5050	14
Danilo	B/21	OxA-15680	Charred grain	*Triticum monococcum*	5987 +/-35	4985-4785	11
Pokrovnik PK-46	A/ 3+4	UCIAMS -140255; PSU-6005	Bone	*Ovis/Capra*	6075 +/-25	5055-4910	
Pokrovnik PK-47	C/2	UCIAMS -140256; PSU-6006	Bone	*Bos taurus*	6105 +/-25	5206-4942	
Pokrovnik	D/3	OxA-17223	Charred grain	*Triticum dicoccum*	6170 +/-35	5220-5015	11
Pokrovnik PK-49	D/7	UCIAMS -140257; PSU-6007	Bone	*Bos taurus*	6165 +/-25	5214-5049	
Pokrovnik PK-44	D/9	UCIAMS -106477; PSU-4960	Bone	*Bos taurus*	6280 +/-20	5310-5215	14
Pokrovnik PK-50	D/14	PSUAMS-3463	Bone	*Bos taurus*	6735 +/-30	5715-5576	
Pokrovnik PK-7	D/22	UCIAMS -116205; PSU-5293	Bone	*Bos taurus*	7090 +/-25	6025-5965 (56.3%); 5960-5905 (39.1%)	14
Pokrovnik PK-15	D/23	UCIAMS -119837; PSU-5556	Bone	*Ovis aries*	6975 +/-30	5980-5945 (8.3%); 5920-5760 (87.1%)	14
