# Supplementary material for: Fatty acid specific δ13C values reveal earliest Mediterranean cheese production 7,200 years ago
Source: PLoS One. 2018 Sep 5;13(9):e0202807. doi: 10.1371/journal.pone.0202807 (PMC6124750; doi:10.1371/journal.pone.0202807)
Supplement: S2 Table — EN = Early Neolithic; MN = Middle Neolithic. Note: Pokrovnik Trench D level 2 and 3 are from one chronological stratum; Pokrovnik Trench D levels 5, 6, 7, and 8 are all from one chronological stratum [16]. Radiocarbon dates were calibrated with OxCal 4.2 [72]. (PDF) [file pone.0202807.s002.pdf]

S2 Table. List of pottery samples and biomarker data. EN = Early Neolithic; MN = Middle Neolithic. Note: Pokrovnik Trench D level 2 and 3 are from one chronological stratum; Pokrovnik Trench D levels 5, 6, 7, and 8 are all from one chronological stratum [see 16]. Radiocarbon dates were calibrated with OxCal 4.2 [73].

Residue Sample #	Site	Trench	Level	Ceramic Type	$\delta^{13}C_{16:0}$	$\delta^{13}C_{18:0}$	Residue type	Cultural Phase	Associated Radiocarbon Sample #	Date BP	Date cal. BC
1	Danilo	A	29	Danilo fine ware	-29.2	-30.4	meat	MN			
2	Danilo	A	35	Figulina	-29.2	-33.4	milk	MN			
3	Danilo	A	30	Danilo fine ware	-29.4	-30.9	meat	MN			
4	Danilo	A	30	Danilo fine ware	-28.7	-31.7	meat	MN			
5	Danilo	A	30	Figulina	-29.4	-34.4	milk	MN			
6	Danilo	A	20	Danilo fine ware	-28.4	-30.4	meat	MN			
8	Danilo	A	30	Figulina			no yield	MN			
9	Danilo	A	29	Danilo fine ware			no yield	MN			
10	Danilo	A	28	Danilo fine ware			no yield	MN			
11	Danilo	A	28	Danilo fine ware			no yield	MN			
12	Danilo	A	20	Danilo fine ware			no yield	MN			
13	Danilo	A	20	Danilo fine ware			no yield	MN			
14	Danilo	A	20	Danilo coarse ware			no yield	MN			
15	Danilo	A	30	Danilo coarse ware			no yield	MN			

16	Danilo	A	30	Danilo coarse ware			no yield	MN			
17	Danilo	A	35	Danilo fine ware			no yield	MN			
18	Danilo	A	36	Rhyton	-26.6	-28.3	cheese	MN	OxA-17197	6121 +/-37	5210-4955
19	Danilo	B	25	Rhyton	-28.4	-30.8	meat	MN			
20	Danilo	B	21	Rhyton	-26.9	-29.2	cheese	MN	OxA-15680	5987 +/-35	4985-4785
21	Pokrovnik	D	7	Rhyton	-27.1	-28.5	cheese	MN	UCIAMS-140257; PSU-6007	6165 +/-25	5214-5049
22	Pokrovnik	D	6	Figulina	-30.1	-34.5	milk	MN	UCIAMS-140257; PSU-6007	6165 +/-25	5214-5049
23	Pokrovnik	D	7	Figulina	-30.1	-33.8	milk	MN	UCIAMS-140257; PSU-6007	6165 +/-25	5214-5049
24	Pokrovnik	D	7	Danilo coarse ware	-28.7	-30.5	meat	MN	UCIAMS-140257; PSU-6007	6165 +/-25	5214-5049
25	Pokrovnik	D	7	Danilo coarse ware	-30.2	-31.2	meat	MN	UCIAMS-140257; PSU-6007	6165 +/-25	5214-5049
26	Pokrovnik	D	7	Danilo fine ware	-30.2	-33.3	milk	MN	UCIAMS-140257; PSU-6007	6165 +/-25	5214-5049
27	Pokrovnik	D	7	Danilo fine ware	-31.4	-32.1	fish	MN	UCIAMS-140257; PSU-6007	6165 +/-25	5214-5049

28	Pokrovnik	D	7	Danilo fine ware	-31.2	-31.7	fish	MN	UCIAMS-140257; PSU-6007	6165 +/-25	5214-5049
29	Pokrovnik	D	8	Danilo fine ware	-28.2	-32.5	meat/ milk	MN	UCIAMS-140257; PSU-6007	6165 +/-25	5214-5049
30	Pokrovnik	D	8	Danilo fine ware	-31.0	-32.7	fish	MN	UCIAMS-140257; PSU-6007	6165 +/-25	5214-5049
31	Pokrovnik	D	9	Figulina	-29.4	-34.0	milk	MN	UCIAMS-106477/PSU-4960	6280 +/-20	5310-5215
32	Pokrovnik	D	2	Figulina	-30.4	-34.2	milk	MN	OxA-17223	6170 +/-35	5220-5015
33	Pokrovnik	D	9	Figulina	-29.8	-34.2	milk	MN	UCIAMS-106477/PSU-4960	6280 +/-20	5310-5215
34	Pokrovnik	D	9	Figulina	-29.6	-33.6	milk	MN	UCIAMS-106477/PSU-4960	6280 +/-20	5310-5215
35	Pokrovnik	D	23	Impresso	-28.5	-31.2	meat	EN	UCIAMS-119837/PSU-5556	6975 +/-30	5980-5760
36	Pokrovnik	D	23	Impresso	-30.7	-32.0	fish	EN	UCIAMS-119837/PSU-5556	6975 +/-30	5980-5760
37	Pokrovnik	D	23	Impresso	-30.5	-32.5	fish	EN	UCIAMS-119837/PSU-5556	6975 +/-30	5980-5760
38	Pokrovnik	D	23	Impresso	-29.7	-30.6	meat	EN	UCIAMS-119837/PSU-5556	6975 +/-30	5980-5760 (87.1%)

39	Pokrovnik	D	22	Impresso	-29.2	-31.3	meat	EN	UCIAMS-116205/PSU-5293	7090 +/-25	6025-5905
40	Pokrovnik	D	22	Impresso	-28.9	-30.9	meat	EN	UCIAMS-116205/PSU-5293	7090 +/-25	6025-5905
41	Pokrovnik	D	22	Impresso	-29.8	-31.4	meat	EN	UCIAMS-116205/PSU-5293	7090 +/-25	6025-5905
42	Pokrovnik	D	14	Impresso	-28.3	-33.5	milk	EN			
43	Pokrovnik	D	14	Impresso	-29.5	-32.2	meat	EN			
44	Pokrovnik	D	13	Impresso	-29.1	-31.8	meat	EN			
45	Pokrovnik	A	3+4	sieve	-26.6	-30.1	cheese	MN	UCIAMS-140255/PSU-6005	6075 +/-25	5055-4910
46	Pokrovnik	C	2	sieve	-26.4	-29.9	fermented	MN	UCIAMS-140256/PSU-6006	6105 +/-25	5206-4942
47	Danilo	B	6	sieve	-24.3	-31.9	cheese	MN	UCIAMS-140258/PSU-6008	5900 +/-25	4831-4716
48	Pokrovnik	C	2	sieve			no yield	MN	UCIAMS-140256/PSU-6006	6105 +/-25	5206-4942
