# Supplementary material for: Fatty acid specific δ13C values reveal earliest Mediterranean cheese production 7,200 years ago
Source: PLoS One. 2018 Sep 5;13(9):e0202807. doi: 10.1371/journal.pone.0202807 (PMC6124750; doi:10.1371/journal.pone.0202807)
Supplement: S3 Table — (PDF) [file pone.0202807.s003.pdf]

S3 Table. Average composition of cattle, sheep, and goat milk and products based on data summarized in [76-79].

Species	Dairy Product	Lactose (%)	Proteins (%)	Fat (%)	Reference
Cattle (*Bos taurus*)	Milk	5.42	3.15	3.25	59, 64
	Yogurt	3.56	3.47	3.25	59, 64
	Soft cheese (e.g., cottage cheese)	0.6	11	4.3	59, 64
	Hard cheese (e.g., cheddar)	0.003	25	33	59, 64
Sheep (*Ovis aries*)	Milk	4.88	5.59	6.82	79
	Soft cheese (e.g. feta)	1.8	16.1	20.2	76
	Hard cheese (e.g., Pecorino)	0.2	25.8	32	76
Goat (*Capra hircus*)	Milk	4.52	3.56	4.51	Averaged values of those presented in 79, Table 1
	Soft or whey cheese (e.g., ricotta)	2.9	7	21	77, 79
	Hard cheese	1.4	30	27	78, 79
