# Supplementary material for: Fatty acid specific δ13C values reveal earliest Mediterranean cheese production 7,200 years ago
Source: PLoS One. 2018 Sep 5;13(9):e0202807. doi: 10.1371/journal.pone.0202807 (PMC6124750; doi:10.1371/journal.pone.0202807)
Supplement: S4 Table — (PDF) [file pone.0202807.s004.pdf]

S4 Table. Summary of molecular and isotopic criteria to distinguish among animal and plant fats associated with archaeological artifacts and soils [10, 35, 38, 53, 55, 80-88].

Dietary Resource		*Ruminant Adipose*	*Milk*	*Dairy Product*	*Aquatic Resource*	*Plants*
Saturated Fatty Acids (FAs)[a]	**$C_{15:0}$**	–[d]	+	+	-	+
	**$C_{16:0}$**	++[e]	+++	+++	+++	++
	**$C_{17:0}$**	*var.*[f]	+	+	+	+
	**$C_{18:0}$**	+++[g]	++	++	++	++
	**$C_{19:0}$**	-	+	+	-	+
FA Ratios	**$C_{16:0}/C_{15:0}$**	-	> 1.0	> 1.0	-	*var.*
	**$C_{16:0}/C_{18:0}$**	< 1.0	> 1.0	> 1.0	*var.*	*var.*
Isoprenoid FAs[b]	**$C_{19:0}$br**	+[h]	-	-	++	-
	**$C_{20:0}$br**	+	++	++	+	-
Alkylphenyl FAs	**$C_{18:3}$**	-	-	-	++	-
	**$C_{20:3}$**	-	-	-	++	-
**$\Delta^{13}C_{16:0 - 18:0}$**		> 3.3	< 3.3	*var.*	> 3.3	*var.*
Acyl Carbon	**TAG**[C]	44-52	40-54	40-54	-	-

[a] Major fatty acid (*n*-alkanoic acid) homologues are shown according to chain-length and number of unsaturations; [b] $C_{19:0}$br indicates 2,6,10,14-tetramethylpentadecanoic (pristanic) acid; $C_{20:0}$br indicates 3,7,11,15-tetramethylhexadecanoic (phytanic) acid; [c] TAG indicates *triacylglyercol* and are shown according to acyl chain-length ranges; [d] Not present; [e] Intermediate relative abundance; [f] Variable abundance; [g] High relative abundance; [h] Low relative abundance.
